# Molecular Structure, Electronic Properties, Reactivity (ELF, LOL, and Fukui), and NCI-RDG Studies of the Binary Mixture of Water and Essential Oil of *Phlomis bruguieri*

**DOI:** 10.3390/molecules28062684

**Published:** 2023-03-16

**Authors:** Feride Akman, Azize Demirpolat, Aleksandr S. Kazachenko, Anna S. Kazachenko, Noureddine Issaoui, Omar Al-Dossary

**Affiliations:** 1 Vocational School of Food, Agriculture and Livestock, University of Bingöl, Bingöl 12000, Turkey; 2 School of Non-Ferrous Metals and Materials Science, Siberian Federal University, Pr. Svobodny 79, 660041 Krasnoyarsk, Russia; 3 Siberian Branch, FRC “Krasnoyarsk Scientific Center”, Institute of Chemistry and Chemical Technology, Russian Academy of Sciences, Akademgorodok 50, Bld. 24, 660036 Krasnoyarsk, Russia; 4 Department of Biological Chemistry with Courses in Medical, Pharmaceutical and Toxicological Chemistry, Krasnoyarsk State Medical University, St. Partizan Zheleznyak, Bld. 1, 660022 Krasnoyarsk, Russia; 5 Laboratory of Quantum and Statistical Physics, LR18ES18, Faculty of Sciences, University of Monastir, Monastir 5079, Tunisia; 6 Departement of Physics and Astronomy, College of Science, King Saud University, P.O. Box 2455, Riyadh 11451, Saudi Arabia

**Keywords:** caryophyllene oxide, *β*-pinene, 1,8-cineol, α-cubebene, β-caryophyllene, essential oil, DFT, NCI-RDG, ELF-LOL

## Abstract

Essential oils are volatile oil-like liquids with a characteristic strong smell and taste. They are formed in plants and are then extracted. Essential oils have extremely strong physiological and pharmacological properties, which are used in the medicine, cosmetics, and food industries. In this study, the molecules caryophyllene oxide, *β*-pinene, 1,8-cineol, α-cubebene, and β-caryophyllene, which are the molecules with the highest contents in the essential oil of the plant mentioned in the title, were selected and theoretical calculations describing their interactions with water were performed. Because oil–water mixtures are very important in biology and industry and are ubiquitous in nature, quantum chemical calculations for binary mixtures of water with caryophyllene oxide, β-pinene, 1,8-cineol, α-cubebene, and β-caryophyllene were performed using the density functional theory (DFT)/B3LYP method with a basis of 6–31 G (d, p). Molecular structures, HOMO–LUMO energies, electronic properties, reactivity (ELF, LOL, and Fukui), and NCI-RDG and molecular electrostatic potential (MEP) on surfaces of the main components of *Phlomis bruguieri* Desf. essential oil were calculated and described.

## 1. Introduction

*Phlomis* is a genus belonging to the family Lamiaceae. It is known by the local names “çalba” and “shalba”. The genus *Phlomis* includes more than 100 species of plants and shrubs distributed in Europe, Asia, and North Africa, and 52 taxa, including 6 species, 12 natural hybrids, and 34 endemic taxa, are known to grow in Turkey [1,2]. In traditional Turkish folk medicine, the flowers and/or leaves of *Phlomis* species are widely used as herbal teas, as a tonic, carminative, and appetite stimulant, and for stomach ache. Besides these traditional uses, *Phlomis* plants have been shown to have antidiabetic, ulcerogenic, antimicrobial, anti-inflammatory, antinociceptive, antimutagenic, immunosuppressive, and free radical scavenging properties. Furthermore, phytochemical investigations of members of the *Phlomis* genus have shown that these plants contain iridoids, flavonoids, phenylpropanoids, phenylethanoids, lignans, neolignans, diterpenoids, alkaloids, and essential oils [3].

Significant advances have been made in understanding the pharmacological activities of *Phlomis* species over the past decade, as well as in the identification of various components they contain. *Phlomis* species contain iridoids, flavonoids, phenylpropanoids, phenylethanoids, lignans, neolignans, diterpenoids, alkaloids, and essential oils, according to phytochemical studies [4,5]. Essential oils derived from various *Phlomis* species have been shown to have antibacterial properties against a wide range of pathogenic bacteria. Previous research has shown that members of the *Phlomis* genus have antiparasitic activity [6]. Some *Phlomis* species are used in Anatolian folk medicine for their antidiarrheal, immunosuppressive, antimutagenic, antifebrile, and free radical scavenging properties, in addition to their other known medicinal properties [7,8].

Previously, the essential oil of *P. bruguieri* has attracted the attention of researchers and the essential oil content of *P. bruguieri* plants collected from Iran has been studied. However, this was the first study conducted in Turkey.

The density functional theory (DFT) method is a promising method for the theoretical study of molecules, since it gives accurate results close to experimental ones [9,10]. DFT is used to study molecular geometry and electron density, which are necessary to obtain data describing molecular properties in a short time [11].

Various functions are used to study simple organic substances, but the B3LYP method, the most famous hybrid model of the density functional theory, which includes the Hartree–Fock variation, gradient variation correction, gradient correlation correction, local variation, and local correlation, has found the most widespread use [12].

The DFT method has also been used to study the components of essential oils. Density functional theory calculations for the monomeric substances of an essential oil allow us to draw conclusions about their molecular properties, charge distribution, chemical activity, and other important characteristics. In [13], the authors investigated the components of the essential oil of *Aethionema sancakense*. In [14], the authors studied the main components of *Lippia sidoides* essential oils. In [15], the authors studied the essential oils of *Piper cernuum Vell* and *Piper rivinoides Kunth*. It should be noted that the authors of these studies chose four to five main components, which were further investigated using theoretical methods. In addition, in these studies, pure substances were investigated as opposed to mixtures, which introduces some inaccuracies when comparing theoretical and experimental data.

In this work, we studied the extracts of *Phlomis bruguieri* Desf. using experimental and theoretical methods. Since the main components of the essential oil of *Phlomis bruguieri* Desf. were caryophyllene oxide, β-pinene, 1,8-cineol, α-cubebene, and β-caryophyllene, we chose these substances for a comprehensive study. Molecular and electronic properties, Fukui functions, and molecular electrostatic potential (MEP) values of surfaces were characterized and RDG analysis was carried out.

## 2. Results

### 2.1. Experimental Study

The plant material used in the study was collected from the natural environment during the flowering period of the plant and dried in the shade. The oil obtained from the plants was extracted using the hydrodistillation method. The oil obtained by hydrodistillation of the air-dried plant materials was injected into a GC/GC-MS device after the distillation process was completed. The retention indices of the obtained components were used as reference points to calculate the retention indices and the chemical compounds, retention times, and mass spectra of the essential oil were determined.

In the analysis of the *P. bruguieri* essential oil, qualitative and quantitative differences were found. In the essential oil of *P. brugueri*, 54 components were identified, representing 100% of the oil. The yield of essential oil was 0.5% (*v*/*w*) of yellowish oil. β-pinene (9.63%), 1,8-cineol (8.64%), α-cubebene (8.89%), β-caryophyllene (8.28%), and caryophyllene oxide (10.56%) were found to be the major essential oil components of the *P. brugueri* aerial part structures. The major constituents obtained from the essential oil of *P. brugueri* plants collected from an Iranian region were germacrene D (23.6%), 4-hydroxy-4-methyl-2-pentanone (15.0%), α-pinene (6.8%), and β-caryophyllene (6.7%) [16]. In another study, caryophyllene oxide (16.3%), γ-muurolene (15.5%), α-selinene (7.1%), and cis-calamene (6.7%) were detected in the analysis of essential oil from *P. bruguieri* plants collected from Iran [17]. In our study, the content of germacrene D was found to be very low. While *α*-pinene (2.76%), β-caryophyllene (8.28%), and caryophyllene oxide (10.56%) were found, 4-hydroxy-4-methyl-2-pentanone was not among the results of our study of *P. bruguieri.* It can be concluded that this difference was due to the location of the plant, the climate, and the time of collection.

### 2.2. Theoretical Study

#### 2.2.1. HOMO–LUMO Analysis

Noncovalent interactions in a binary mixture play an important role in determining the mixture’s thermodynamic properties and reactivity [18,19]. Mixtures of simple substances with water are subjects of active study both experimentally and theoretically [20,21].

The interactions between water and organic substances (in a ratio of 1:1) are subjects of active experimental and theoretical study. Thus, data are available for interactions between single water molecules and furan [22], imidazole [23], pyridine [24], corannulene [25], benzene [26], chlorotrifluoroethylene [27], and many others [28,29].

Studies of the components of essential oils using experimental and theoretical methods play an important role in understanding their impacts and potential uses [13,30,31]. First, after optimizing the structures, we performed a HOMO–LUMO analysis.

**Figure 1:**
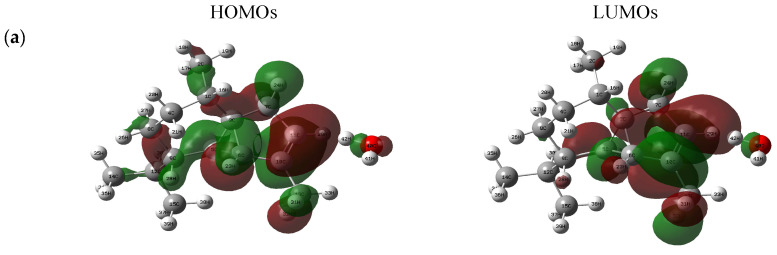

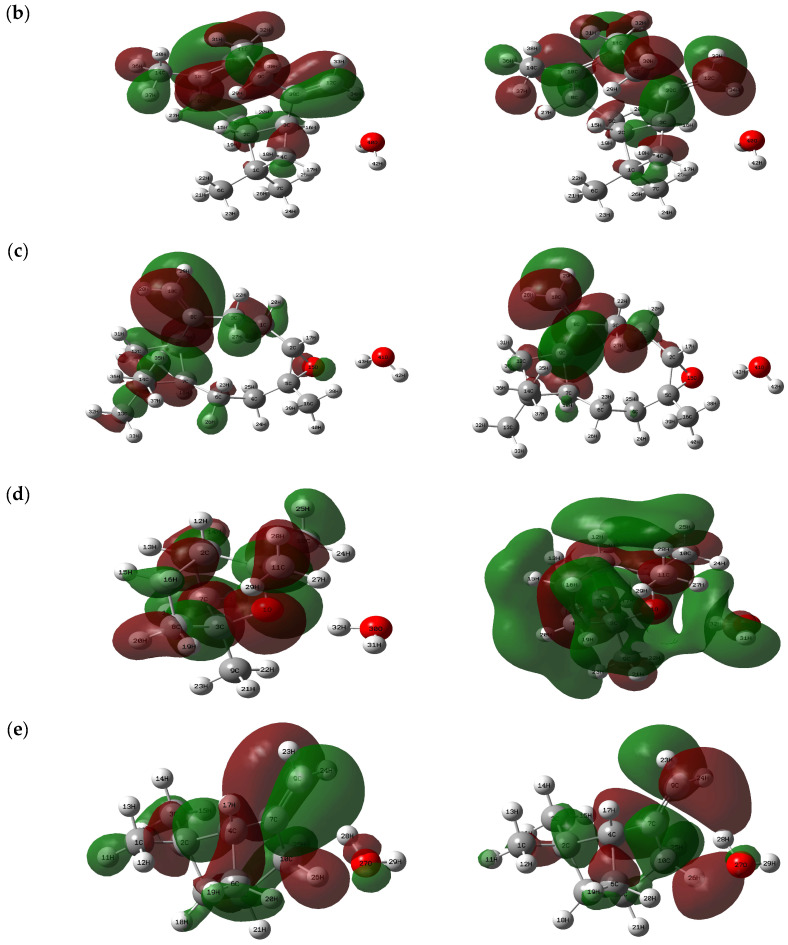
HOMO–LUMO plots of the binary mixtures (α-cubebene–water (**a**), β-caryophyllene–water (**b**), caryophyllene oxide–water (**c**), 1,8-cineole–water (**d**), and β-pinene–water (**e**)).

The highest occupied molecular orbital and the lowest unoccupied molecular orbital are very important parameters in quantum chemistry [32]. This information is also used along with the boundary electron density to predict the most reactive positions in various systems [33]. The highest occupied molecular orbital and the lowest unoccupied molecular orbital are the main orbitals involved in chemical stability [34]. The energy difference between the HOMO and LUMO orbitals is called the energy gap, which is an important stability characteristic of structures. In Figure 1, green and red colors represent the positive and negative phases, respectively. The energy values of the HOMO (donor) and LUMO (acceptor), and their energy gap, reflect the chemical activity of a molecule [35,36]. In general, an atom with a higher HOMO density should have a greater ability to donate an electron, while an atom with a higher occupied LUMO density should have a greater tendency to gain an electron [37]. Three-dimensional plots of the HOMO and LUMO orbitals for the binary mixtures of the essential oil components with water are indicated in Figure 1.

HOMO and LUMO energies (E_HOMO_ and E_LUMO_), energy gap (Eg), electronegativity (χ), electron affinity (EA), softness (ζ), chemical potential (μ), ionization potential (IP), hardness (η), global electrophilicity index (ω), optical softness (σ_o_), maximum charge transfer index (∆N_max_), and nucleophilicity index (N) for the α-cubebene–water, β-caryophyllene–water, caryophyllene oxide–water, 1,8-cineole–water, and β-pinene–water mixtures were computed using the B3LYP/6–31 G (d,p) basis set. The data are given in Table 1. These quantum chemical descriptors, determined using the HOMO and LUMO energies, can be defined as follows [38]:

(1) $$\mathrm{IP}=-E_{\mathrm{HOMO}}$$

(2) $$\mathrm{EA}=-E_{\mathrm{LUMO}}$$

(3) Eg= IP−EA

(4) χ=12(EA+IP)

(5) η=12(IP−EA)

(6) $$\zeta =\frac{2}{\left(\mathrm{IP}-\mathrm{EA}\right)}$$

(7) μ=−12(EA+IP)

(8) $$\omega =\frac{\mu^{2}}{2\eta }$$

(9) $$\Delta N_{\max}=-\frac{\mu }{\eta }$$

(10) $$N=\frac{1}{\omega }$$

(11) $$\sigma_{o}=\frac{1}{\mathrm{Eg}}$$

According to the data given in Table 1, for all the studied substances, E_HOMO_ fell within the range of −5.8 to −6.7 eV, while E_LUMO_ fell within the range of 0.4 to 1.7 eV. Among all the studied substances, the highest E_gap_ value (about 8.3 eV) was observed for 1,8-cineole–water, which indicated the greatest stability among the studied substances. The lowest E_gap_ value (about 6.47 eV) was observed for α-cubebene–water.

According to the data given in Table 1, the introduction of a water molecule led to changes in the values of the energy gap and other physical parameters. Thus, for α-cubebene, a decrease in the size of the energy gap was observed when a water molecule was introduced into the system. A similar change was also observed for β-pinene. For β-caryophyllene, caryophyllene oxide, and 1,8-cineole, the observations were different. For these substances, the introduction of a water molecule increased the size of the energy gap.

The data obtained partially agree with the literature data. Thus, in [39], for pure α-cubebene, the observed values of E_HOMO_, E_LUMO_, and Egap corresponded to −5.69, 0.90, and 6.59, respectively; for a mixture with water, these values were −5.96, 0.51, and 6.47 eV, respectively. When comparing the data given in [39] and Table 1, it is apparent that the introduction of one water molecule into a system led to changes in most physical parameters. For example, the value of ω for pure α-cubebene was 0.44 [39], and for its binary mixture in water was 1.1481.

The introduction of a water molecule also affected other physical parameters (Table 1). In general, there was a correlation with the previously described phenomenon, in that those parameters that increased for α-cubebene and β-pinene when a water molecule was introduced into the system decreased for β-caryophyllene, caryophyllene oxide, and 1,8-cineole, and vice versa.

Thus, it can be assumed that the introduction of water into these systems slightly changed the values of the energy gap for the substances under study. The data obtained were consistent with the results given in [20,21].

#### 2.2.2. MEP Analysis

**Figure 2:**
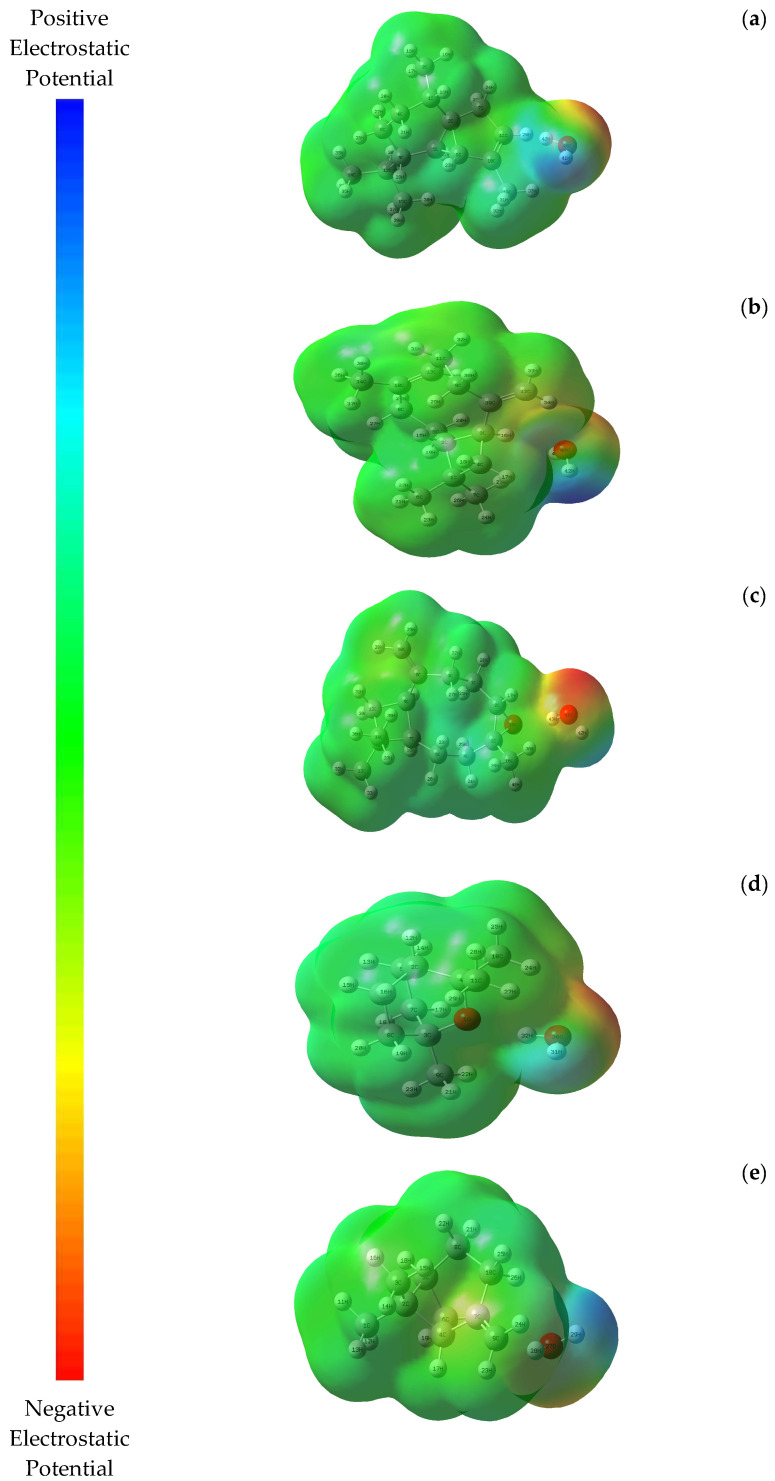
MEP surface maps of the binary mixtures (α-cubebene–water (**a**), β-caryophyllene–water (**b**), caryophyllene oxide–water (**c**), 1,8-cineole–water (**d**), and β-pinene–water (**e**)).

The net electrostatic effect produced by the overall charge distribution (electrons + nuclei) of a molecule at a point in space around it is known as the molecular electrostatic potential. It relates the total charge distribution to dipole moments, partial charges, electronegativity, and the site of chemical activity of the molecule [40]. In Figure 2, different electrostatic potential values on the surface are represented by different colors: red represents the areas with the most negative electrostatic potential, blue represents the areas with the most positive electrostatic potential, and green represents areas with zero potential.

The most positive and negative regions, represented by blue and red colors on the MEP map, are the preferred locations for nucleophilic and electrophilic attacks, respectively [41]. Such maps are very useful when studying molecular structure and physicochemical characteristics because they use color grading to represent areas of positive, negative, and neutral electrostatic potential [42].

In all the molecules we studied, the electrostatic potential was primarily limited to oxygen, which is a reactive region for electrophilic attack (Figure 2). It should be noted that the red region was located above the water oxygen in the binary mixtures under study, and the blue region was located mainly above the hydrogen atom in the water (Figure 2).

#### 2.2.3. Energy of Population Density of States

**Figure 3:**
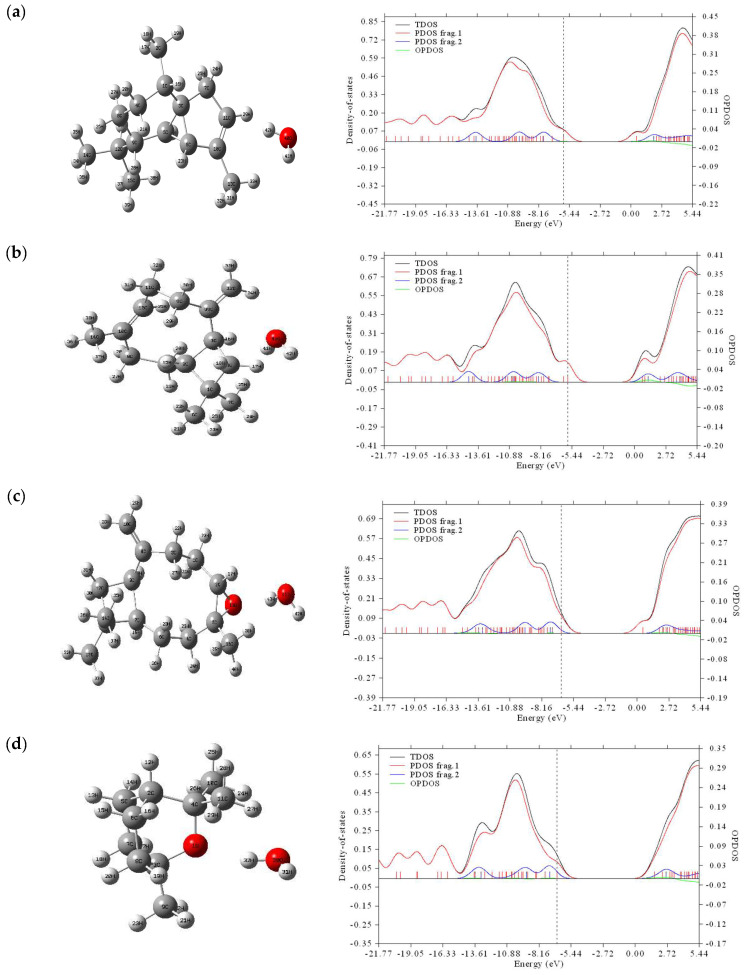

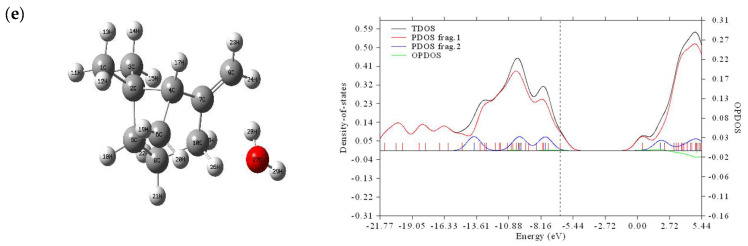
TDOS, PDOS, and OPDOS plots of the binary mixtures (α-cubebene–water (**a**), β-caryophyllene–water (**b**), caryophyllene oxide–water (**c**), 1,8-cineole–water (**d**), and β-pinene–water (**e**)) (frag. 1-pure molecule; frag. 2-binary mixture with water).

Since nearby orbitals in a boundary region may have quasi-degenerate energy levels, using only the HOMO and LUMO to describe the boundary orbitals may not be complete enough [43]. The DOS spectrum explains the contribution of electrons to the conduction and valence bands. The spectra of the investigated binary mixtures, which illustrate how many states are available at different energy levels, are presented in Figure 3.

A virtual orbital is also known as an acceptor orbital because it is not occupied. On the other hand, a filled orbital is known as a donor orbital. DOS is positive for binding interactions, negative for antibinding interactions, and zero for no binding interaction [44].

For visual representation of MO compositions and their contributions to chemical bonding, total (TDOS), partial (PDOS), and overlap (OPDOS) population densities of states for α-cubebene, β-caryophyllene, caryophyllene oxide, 1,8-cineole, and β-pinene and their binary mixtures with water are plotted in Figure 3.

According to the data shown in Figure 3, the TDOS (black) and PDOS frag. 1 (red) curves had the same shape and size for all the studied substances from −21.77 to −15.57 eV. In the range of −15.56 to 5.44 eV, these curves had similar shapes, but different sizes, while the TDOS curve, as a rule, was above the PDOS frag. 1 curve. It should be noted that PDOS frag. 2 (blue) had very different shapes and sizes from PDOS frag. 1. Thus, the introduction of water molecules strongly affected the TDOS, PDOS, and OPDOS data of the substances under study.

The energy gaps observed in the DOS and HOMO–LUMO spectra for the studied substances were consistent with each other (Table 2).

According to the data shown in Table 2, the introduction of a water molecule led to a shift in the center of the PDOS towards a lower value for all the substances under study. Thus, for α-cubebene, a shift from −6.718485 to −5.640946 eV was observed when a water molecule was added.

#### 2.2.4. NCI-RDG Analysis

RDG is an efficient method used to study noncovalent interactions in real space based on electron density and its derivatives [45]. This approach has been shown to be able to distinguish between hydrogen bonds, van der Waals interactions, and steric repulsion in small molecules and molecular complexes. To calculate the RDG values, we used the following equation:

(12) $$\mathrm{RDG}\left(r\right)=\frac{1}{2{\left(3\pi^{2}\right)}^{1/3}}\frac{\left|\nabla \rho \left(r\right)\right|}{\rho {\left(r\right)}^{4/3}}$$

By analyzing the low-density gradient, regions of low electron content, which are responsible for weak interactions, can be confirmed; similarly, the high-density gradient value is used to identify strong interactions. Plots of RDG versus ρ (low-gradient peak-density values) seem to be an indicator of the strength of an interaction. The product of the electron density ρ and the sign of the second eigenvalues λ_2_ has been proposed as a tool to distinguish between a wide range of interaction types. The sign λ_2_ is a critical parameter used to distinguish between bound (λ_2_ < 0) and noncoupling (λ_2_ > 0) interactions. On the basis of (λ_2_)ρ, interactions can be divided into three types. The first is an attraction (hydrogen bond or dipole–dipole) and is characterized by negative sign values (λ_2_)ρ. The second assumes a nonbinding interaction of a strong repulsion type, which is confirmed by large and positive values of the sign (λ_2_)ρ. The third type, namely weak interactions (van der Waals interactions), is associated with values close to zero.

**Figure 4:**
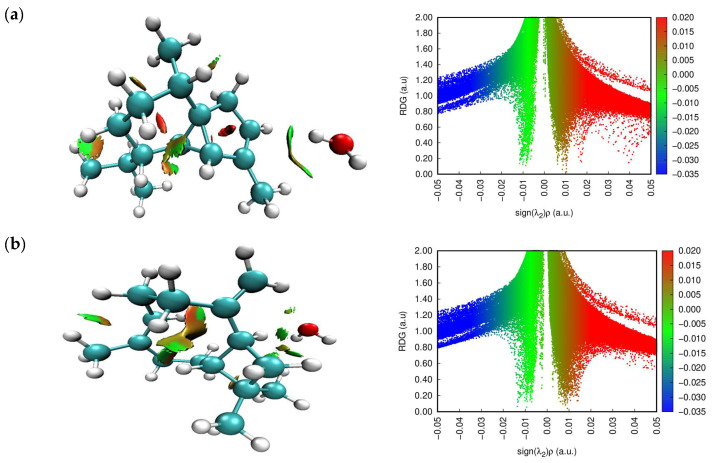

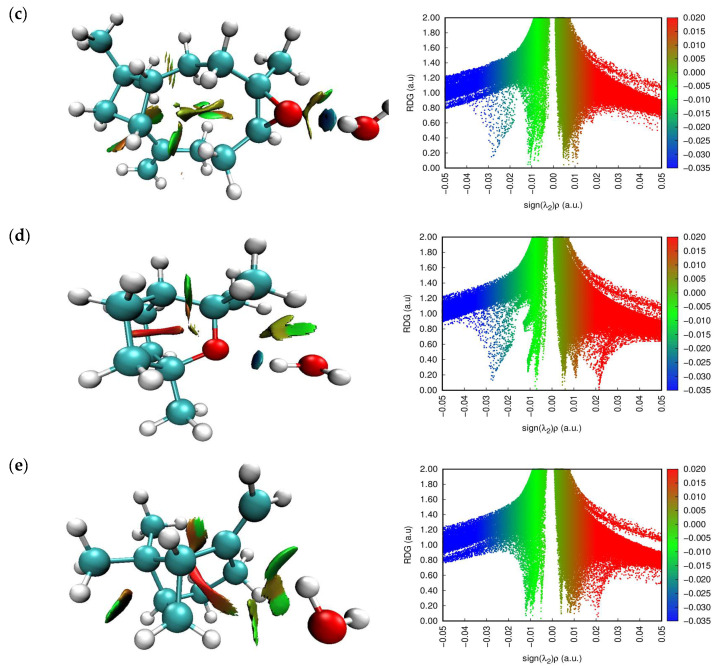
NCI (**left**) and RDG (**right**) plots of the binary mixtures (α-cubebene–water (**a**), β-caryophyllene–water (**b**), caryophyllene oxide–water (**c**), 1,8-cineole–water (**d**), and β-pinene–water (**e**)).

Figure 4 shows the predicted RDG plots for the test substances. The interactions between water and α-cubebene, β-caryophyllene, caryophyllene oxide, 1,8-cineole, and β-pinene were described by different types of interactions. The first—red color—was typical for all the studied substances, indicating a strong repulsion within the system. The second—green—showed the van der Waals interactions. The third—blue color—corresponded to strong hydrogen bond interactions and was clearly observed between the water molecules and caryophyllene oxide and 1,8-cineole.

#### 2.2.5. ELF and LOL Analyses

**Figure 5:**
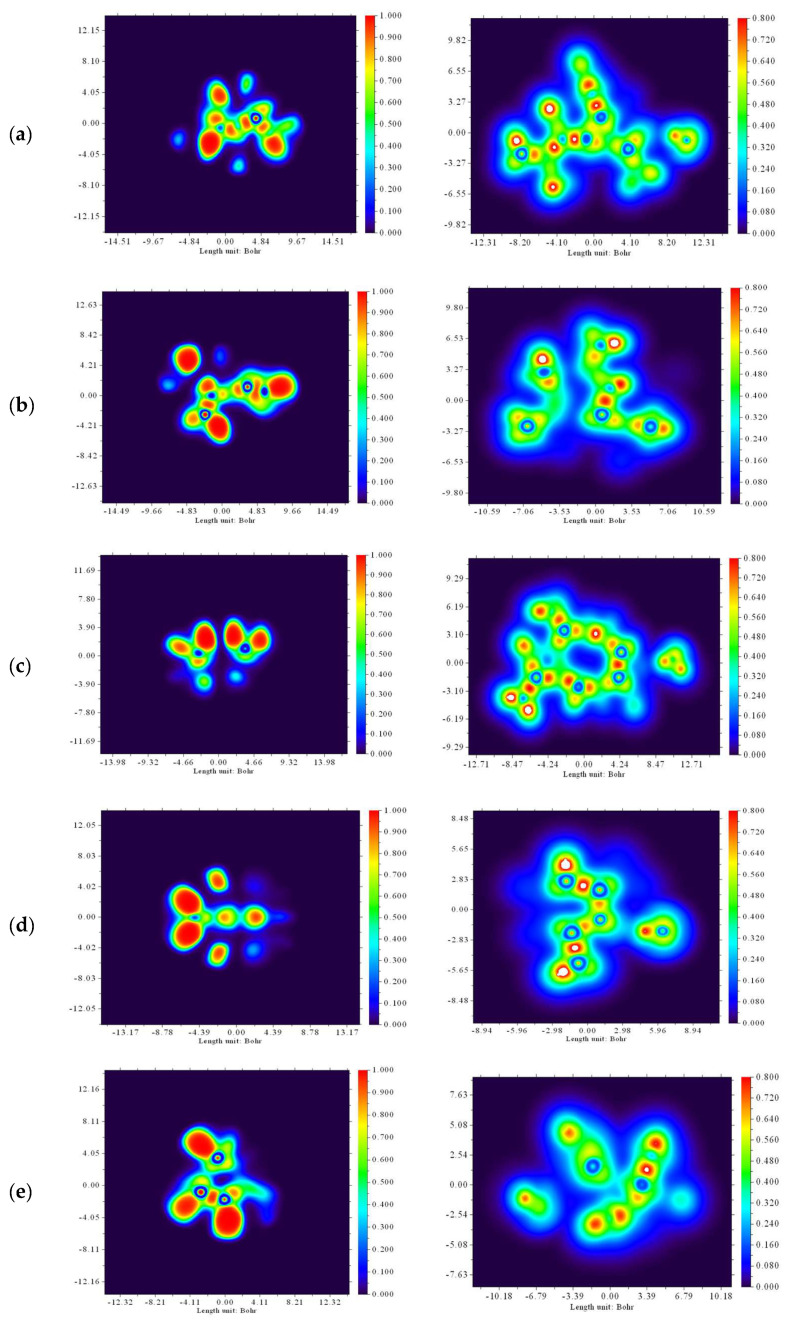
Electron localization function (ELF) maps (**left**) and localized orbital locator (LOL) maps (**right**) of the binary mixtures (α-cubebene–water (**a**), β-caryophyllene–water (**b**), caryophyllene oxide–water (**c**), 1,8-cineole–water (**d**), and β-pinene–water (**e**)).

Surface analysis based on covalent bonding was performed using an electron localization function (ELF) with a bump map and LOL maps. A bump map with a large or narrowed peak area describes the electronic environment of an atom. They offer a valuable opportunity to identify pairs of electrons on the molecular surface [46,47]. ELF and LOL have similar chemical mapping because they depend on the kinetic energy density. However, ELF arises from the electron pair density; LOL simply exhibits a gradient of localized orbitals and is used when localized orbitals overlap [46]. Images of the studied substances in ELF and LOL formats are shown on the color tint map and contour maps of the hydrogen bond region in Figure 5. The ELF map was built in the range of 0.0–1.0; however, delocalized electrons are projected in a reduced range (<0.5) [48,49]. If an electron position dominates in the electron density, then the LOL phase reaches the upper region (>0.5) [50,51]. Due to the presence of a covalent bond or nuclear layer, a high position of electrons is created in this region, as demonstrated by its high value in this region. The electron cloud was delocalized around some carbon atoms in the studied substances, which is represented by blue areas. Critical points and their trajectories, chemical bonding, and chemically significant regions (red and orange) are clearly shown on the ELF map, localized mainly around hydrogen atoms and present in all substances under study. It should be noted that the central regions of some hydrogen atoms in all the studied substances were white, which indicates that the electron density exceeded the upper limit of the color scale (0.80) (Figure 5).

#### 2.2.6. Fukui Analysis

The condensed Fukui functions are determined to characterize the regioselectivity of an atom ‘r’ in a molecule. For this purpose, the Fukui functions (f^+^ (r), f^−^ (r), f^0^ (r), and Δf(r)) were calculated using Multiwfn [52]. The Fukui functions described by Kolandaivel et al. can be calculated using the following equations [53]:

f^-^ (r)= q_(N)_ (r) − q_(N−1)_ (r) (for electrophilic attack)

f^+^ (r) = q_(N+1)_ (r) − q_(N)_ (r) (for nucleophilic attack)

f^0^ (r) = 1/2 [q_(N+1)_ (r) − q_(N−1)_ (r)] (for radical attack)

where q_(N)_ (r) is the atomic charge on site ‘r’ in a neutral system; q_(N−1)_ (r) is the atomic charge on site ‘r’ in a cationic system; and q_(N+1)_ (r) is the atomic charge on site ‘r’ in an anionic system. Additionally, the dual identifier Δf(r) is the difference between nucleophilic and electrophilic attacks at a specific site and can be calculated according to the following equation [54,55,56]:

Δf(r) = f^+^ (r) − f^−^ (r) (13)

If this value is negative, the particular area can be considered an electrophilic attack site. If this value is positive, then it may be a nucleophilic attack site.

The Fukui functions of the molecules (a–d) were calculated using Multiwfn based on the energies calculated using the B3LYP/6–31 G (d,p) method and are listed in Table 3 and Table 4.

According to Table 3, the descriptor values for water with α-cubebene (a) and β-caryophyllene (b) indicated that the sites of nucleophilic attack were 4C, 10C, 11C, 13C, 21H, 25H, 28H, 29H, 31H, 32H, 33H, 40O, 41H, and 42H and 6C, 7C, 15H, 21H, 22H, 24H, 25H, 26H, 30H, 39C, 40O, 41H, and 42H, respectively, while sites of electrophilic attack were on the other atoms of the molecules. According to Table 3, the descriptor values for water in caryophyllene oxide–water (c) indicated that the sites of nucleophilic attack were 3C, 8C, 9C, 10C, 18H, 19H, 21H, 22H, 25H, 27H, 28H, 29H, 30H, 33H, 34H, 36H, 37H, and 38H, while the sites of electrophilic attack were on other atoms of the molecules. According to Table 4, the descriptor values for water with 1,8-cineole (d) and β-pinene (e) indicated that the sites of nucleophilic attack were 2C, 5C, 6C, 9C, 12H, 13H, 14H, 15H,16H, 17H,18H, 19H, 20H, 21H, 22H, 23H, 24H, 25H, 26H, 27H, 28H, 29H, 31H, and 32H and 7C, 10C, 21H, 23H, 24H, 25H, 26H, 27H, 28H, and 29H, respectively, while the sites of electrophilic attack were on the other atoms of the molecules.

## 3. Materials and Methods

### 3.1. Collection of the Plant Material

The plant material used in the study was collected in Turkey. It was collected from its natural habitat in June, which is the flowering period of the plant, and dried in the shade. The plant specimens are preserved at Bingol University.

Sampling location: Turkey, Bingol, Yelesen village, plowed pasture fields. Altitude: 1500–1600 m. Latitude 38.869837, longitude 40.321814.

### 3.2. Isolation of Essential Oil and GC-MS Analysis

The plants used in this study were air-dried. The oil was extracted from the plants using the hydrodistillation method. The air-dried aerial parts (200 g) of the plants were hydrodistilled for three hours in a Clevenger-type apparatus. The organic layer in the collection vial was injected into the GC/GC-MS instrument after the distillation was completed.

GC-MS was used to examine the essential oil. The MS instrument was a Shimadzu GCMS-QP2020 model. The mass range was 40–330 *m*/*z* and the ionization energy was 70 eV. An RXI-5MS capillary column (30 m × 0.25 mm × 0.25 m) was used. The carrier gas was helium, with a constant column flow rate of 1 mL/min. The column oven temperature program was set to a temperature of 40 °C with a hold time of two minutes and a rate of change of 3 °C/min. The final temperature was 240 °C The injection volume was set to 1 l and the mode was set to split (split ratio 1:10 or 1:100). A 3.5 min solvent delay was used for hexane samples. The mass spectrometric parameters were as follows: full scan mode, 20,000 amu/s scan speed, and 50 spectrum sampling frequency. The temperatures at the interface and the ion source were 250 °C and 200 °C, respectively.

Alkanes were used as reference points to calculate the retention indices (RI). The chemical compounds of the essential oils were identified by comparing their retention times (RT) and mass spectra to those reported in MS libraries (Wiley MS library, New York, NY, USA) and the literature (NIST 20 and Wiley Libraries) [57]. A traditional library search ignores retention parameters, and the process consists solely of comparing spectra. When searching libraries in this study, a combination of storage indexes were used, making compound identification easier and more reliable. The device’s retention index spectral libraries were also used in this study. The same analysis method as described for the column library was used for better results.

The identified constituents of the essential oil are listed in Table 5.

### 3.3. Theoretical Study

DFT calculations were performed using Gaussian 09W software [76] and the GausView 0.5 program [77]. The optimized molecular structures of the binary mixtures of α-cubebene, β-caryophyllene, caryophyllene oxide, 1,8-cineole, and β-pinene with water were calculated with the 6–31 G(d,p) basis set using Becke’s three-parameter hybrid functional (B3) [78] and the Lee–Yang–Parr (LYP) correlation function [79]. Molecular electrostatic potential surface maps and HOMO–LUMO plots were plotted using the same method. Using the Multiwfn software [80], total density of state (TDOS), partial density of state (PDOS), and overlap population density of state (OPDOS) analyses were carried out for the binary mixtures. Furthermore, scatter plots of the reduced density gradients (RDGs) and noncovalent interactions (NCI) were constructed using Multiwfn software and visualized using VMD software [81]. Finally, ELF, LOL, and Fukui functions were calculated using Multiwfn 3.8 software.

## 4. Conclusions

Essential oils have a wide range of beneficial properties. In this work, we isolated the essential oil from *Phlomis bruguieri*. It was found that caryophyllene oxide, β-pinene, 1,8-cineol, α-cubebene, and β-caryophyllene showed the maximum yields among the substances found in the essential oil of this plant. For these substances, calculations describing their mixtures with water were carried out, since this information also has industrial significance. It was shown that the introduction of water into these systems affected the electronic properties of the molecules, including changing the size of the energy gaps. Molecular structure, electronic properties, reactivity (ELF, LOL, and Fukui), and NCI-RDG data were obtained for the tested substances. The energy gaps observed in the DOS and HOMO–LUMO spectra for the studied substances were comparable and consistent with each other. The results obtained in this work can be applied in various fields from aromatherapy [82] and medicine [83] to encapsulation and food chemistry [84,85]. In addition, the theoretical data obtained in this work may help others to understand the noncovalent interactions of some components of essential oils with water.

## Figures and Tables

**Table 1:** Various physical parameters of the binary mixtures (α-cubebene–water (a), β-caryophyllene–water (b), caryophyllene oxide–water (c), 1,8-cineole–water (d), and β-pinene–water (e)) and pure molecules (without water) (*).

Parameter	Value (eV)									
	a	a *	b	b *	c	c *	d	d *	e	e *
E_HOMO_	−5.9623	−5.070	−5.8034	−5.9256	−6.4801	−6.4080	−6.6358	−6.2420	−6.5218	−6.2352
E_LUMO_	0.5102	0.8678	0.6860	0.5170	0.4882	0.5494	1.6436	1.8561	0.4337	0.7780
Egap	6.4725	6.5748	6.4894	6.4426	6.9683	6.9574	8.2793	8.0981	6.9555	7.0132
IP	5.9623	5.7070	5.8034	5.9256	6.4801	6.4080	6.6358	6.2420	6.5218	6.2352
EA	−0.5102	−0.8678	−0.6860	−0.5170	−0.4882	−0.5494	−1.6436	−1.8561	−0.4337	−0.7780
χ	2.7260	2.4196	2.5587	2.7043	2.9960	2.9293	2.4961	2.1930	3.0440	2.7286
μ	−2.7260	−2.4196	−2.5587	−2.7043	−2.9960	−2.9293	−2.4961	−2.1930	−3.0440	−2.7286
η	3.2363	3.2874	3.2447	3.2213	3.4841	3.4787	4.1397	4.0491	3.4778	3.5066
ζ	0.3090	0.3042	0.3082	0.3104	0.2870	0.2875	0.2416	0.2470	0.2875	0.2852
ω	1.1481	0.8905	1.0089	1.1351	1.2881	1.2333	0.7525	0.5939	1.3322	1.0616
ΔN_max_	0.8423	0.7360	0.7886	0.8395	0.8599	0.8421	0.6030	0.5416	0.8753	0.7781
σ_o_	0.1545	0.1521	0.1541	0.1552	0.1435	0.1437	0.1208	0.1235	0.1438	0.1426
N	0.8710	1.1230	0.9912	0.8810	0.7763	0.8108	1.3288	1.6839	0.7507	0.9420

**Table 2:** Energy of population densities of states for the binary mixtures (α-cubebene–water (a), β-caryophyllene–water (b), caryophyllene oxide–water (c), 1,8-cineole–water (d), and β-pinene–water (e)) (frag. 1—pure molecule; frag. 2—binary mixture with water).

Molecules	Center of TDOS (eV)	Center of PDOS for Frag. 1 (eV)	Center of PDOS for Frag. 2 (eV)	Vertical Dash Line (HOMO) (eV)
a	−6.642774	−6.718485	−5.640946	−5.96221
b	−6.827964	−6.925306	−5.642114	−5.80330
c	−6.866536	−6.893760	−6.445586	−6.48017
d	−6.806544	−6.846677	−6.373228	−6.63586
e	−6.629970	−6.759459	−5.494805	−6.52173

**Table 3:** The condensed Fukui functions for the binary mixtures α-cubebene–water (a), β-caryophyllene–water (b), and caryophyllene oxide–water (c).

a					b					c				
Atoms	$f_{r}^{-}$	$f_{r}^{+}$	$f_{r}^{0}$	Δf(r)	Atoms	$f_{r}^{-}$	$f_{r}^{+}$	$f_{r}^{0}$	Δf(r)	Atoms	$f_{r}^{-}$	$f_{r}^{+}$	$f_{r}^{0}$	Δf(r)
1(C)	0.0081	0.0031	0.0056	−0.0051	1(C)	0.0074	0.0062	0.0068	−0.0012	1(C)	0.0138	0.0131	0.0134	−0.0008
2(C)	0.0130	0.0087	0.0108	−0.0043	2(C)	0.0045	0.0020	0.0032	−0.0026	2(C)	0.0084	0.0041	0.0062	−0.0044
3(C)	0.0212	0.0146	0.0179	−0.0066	3(C)	0.0065	0.0051	0.0058	−0.0014	3(C)	0.0150	0.0188	0.0169	0.0038
4(C)	0.0066	0.0066	0.0066	0.0000	4(C)	0.0233	0.0132	0.0182	−0.0101	4(C)	0.0083	0.0063	0.0073	−0.0020
5(C)	0.0558	0.0291	0.0424	−0.0268	5(C)	0.0104	0.0069	0.0087	−0.0035	5(C)	0.0093	0.0039	0.0066	−0.0054
6(C)	0.0535	0.0196	0.0365	−0.0339	6(C)	0.0073	0.0076	0.0075	0.0003	6(C)	0.0096	0.0051	0.0074	−0.0045
7(C)	0.0276	0.0263	0.0270	−0.0013	7(C)	0.0064	0.0113	0.0088	0.0049	7(C)	0.0215	0.0162	0.0189	−0.0053
8(C)	0.0117	0.0078	0.0097	−0.0039	8(C)	0.0178	0.0156	0.0167	−0.0023	8(C)	0.0837	0.1223	0.1030	0.0386
9(C)	0.0128	0.0101	0.0114	−0.0027	9(C)	0.0242	0.0168	0.0205	−0.0073	9(C)	0.0175	0.0208	0.0191	0.0033
10(C)	0.0897	0.1289	0.1093	0.0393	10(C)	0.0941	0.0684	0.0812	−0.0257	10(C)	0.1499	0.1723	0.1611	0.0224
11(C)	0.1358	0.1379	0.1368	0.0021	11(C)	0.0266	0.0182	0.0224	−0.0085	11(C)	0.0144	0.0041	0.0092	−0.0103
12(C)	0.0041	0.0023	0.0032	−0.0018	12(C)	0.1087	0.0828	0.0957	−0.0259	12(C)	0.0132	0.0121	0.0127	−0.0012
13(C)	0.0241	0.0335	0.0288	0.0094	13(C)	0.0908	0.0628	0.0768	−0.0280	13(C)	0.0183	0.0137	0.0160	−0.0046
14(C)	0.0110	0.0085	0.0097	−0.0025	14(C)	0.0228	0.0193	0.0210	−0.0035	14(C)	0.0104	0.0088	0.0096	−0.0016
15(C)	0.0046	0.0030	0.0038	−0.0015	15(H)	0.0012	0.0030	0.0021	0.0018	15(O)	0.0565	0.0209	0.0387	−0.0356
16(H)	0.0138	0.0094	0.0116	−0.0043	16(H)	0.0116	0.0075	0.0095	−0.0041	16(C)	0.0108	0.0072	0.0090	−0.0036
17(H)	0.0097	0.0081	0.0089	−0.0016	17(H)	0.0208	0.0172	0.0190	−0.0036	17(H)	0.0145	0.0112	0.0129	−0.0033
18(H)	0.0239	0.0202	0.0221	−0.0037	18(H)	0.0126	0.0122	0.0124	−0.0004	18(H)	0.0180	0.0366	0.0273	0.0186
19(H)	0.0085	0.0061	0.0073	−0.0024	19(H)	0.0217	0.0168	0.0193	−0.0050	19(H)	0.0418	0.0632	0.0525	0.0215
20(H)	0.0214	0.0185	0.0200	−0.0030	20(H)	0.0094	0.0064	0.0079	−0.0029	20(H)	0.0189	0.0179	0.0184	−0.0010
21(H)	0.0081	0.0082	0.0082	0.0001	21(H)	0.0108	0.0108	0.0108	0.0000	21(H)	0.0066	0.0095	0.0080	0.0030
22(H)	0.0213	0.0118	0.0165	−0.0095	22(H)	0.0039	0.0066	0.0052	0.0027	22(H)	0.0183	0.0205	0.0194	0.0022
23(H)	0.0373	0.0320	0.0346	−0.0053	23(H)	0.0160	0.0145	0.0153	−0.0015	23(H)	0.0054	0.0042	0.0048	−0.0012
24(H)	0.0480	0.0382	0.0431	−0.0098	24(H)	0.0161	0.0176	0.0169	0.0015	24(H)	0.0169	0.0161	0.0165	−0.0008
25(H)	0.0371	0.0476	0.0423	0.0104	25(H)	0.0025	0.0088	0.0056	0.0063	25(H)	0.0052	0.0072	0.0062	0.0020
26(H)	0.0215	0.0207	0.0211	−0.0008	26(H)	0.0111	0.0151	0.0131	0.0040	26(H)	0.0235	0.0178	0.0207	−0.0057
27(H)	0.0123	0.0097	0.0110	−0.0026	27(H)	0.0228	0.0200	0.0214	−0.0028	27(H)	0.0245	0.0335	0.0290	0.0089
28(H)	0.0185	0.0191	0.0188	0.0006	28(H)	0.0365	0.0277	0.0321	−0.0089	28(H)	0.0435	0.0589	0.0512	0.0154
29(H)	0.0486	0.0567	0.0526	0.0081	29(H)	0.0202	0.0151	0.0176	−0.0051	29(H)	0.0471	0.0634	0.0552	0.0164
30(H)	0.0065	0.0055	0.0060	−0.0010	30(H)	0.0329	0.0352	0.0341	0.0023	30(H)	0.0205	0.0280	0.0243	0.0075
31(H)	0.0332	0.0444	0.0388	0.0112	31(H)	0.0284	0.0260	0.0272	−0.0024	31(H)	0.0121	0.0112	0.0116	−0.0009
32(H)	0.0358	0.0471	0.0415	0.0113	32(H)	0.0304	0.0196	0.0250	−0.0108	32(H)	0.0209	0.0189	0.0199	−0.0020
33(H)	0.0215	0.0255	0.0235	0.0040	33(H)	0.0362	0.0353	0.0358	−0.0008	33(H)	0.0161	0.0172	0.0166	0.0011
34(H)	0.0171	0.0141	0.0156	−0.0030	34(H)	0.0367	0.0302	0.0335	−0.0065	34(H)	0.0136	0.0152	0.0144	0.0016
35(H)	0.0087	0.0083	0.0085	−0.0004	35(H)	0.0266	0.0214	0.0240	−0.0052	35(H)	0.0004	−0.0028	−0.0012	−0.0032
36(H)	0.0093	0.0085	0.0089	−0.0008	36(H)	0.0356	0.0280	0.0318	−0.0076	36(H)	0.0193	0.0201	0.0197	0.0008
37(H)	0.0171	0.0148	0.0160	−0.0023	37(H)	0.0316	0.0271	0.0293	−0.0046	37(H)	0.0164	0.0185	0.0174	0.0021
38(H)	−0.0042	−0.0061	−0.0051	−0.0019	38(H)	0.0175	0.0155	0.0165	−0.0020	38(H)	0.0084	0.0090	0.0087	0.0006
39(H)	0.0081	0.0069	0.0075	−0.0012	39(C)	0.0472	0.0486	0.0479	0.0014	39(H)	0.0108	0.0076	0.0092	−0.0032
40(O)	0.0247	0.0344	0.0295	0.0097	40(O)	−0.0017	0.0469	0.0226	0.0486	40(H)	0.0149	0.0130	0.0139	−0.0019
41(H)	0.0135	0.0322	0.0228	0.0187	41(H)	0.0054	0.0677	0.0365	0.0623	41(O)	0.0653	0.0114	0.0384	−0.0539
42(H)	−0.0008	0.0182	0.0087	0.0190	42(H)	0.0053	0.0633	0.0343	0.0580	42(H)	0.0244	0.0191	0.0217	−0.0053
										43(H)	0.0119	0.0039	0.0079	−0.0080

**Table 4:** The condensed Fukui functions for the binary mixtures 1,8-cineole–water (d) and β-pinene–water (e).

d					e				
Atoms	$f_{r}^{-}$	$f_{r}^{+}$	$f_{r}^{0}$	Δf(r)	Atoms	$f_{r}^{-}$	$f_{r}^{+}$	$f_{r}^{0}$	Δf(r)
1(O)	0.1773	0.0111	0.0942	−0.1661	1(C)	0.0191	0.0154	0.0173	−0.0036
2(C)	0.0158	0.0165	0.0162	0.0006	2(C)	0.0343	0.0132	0.0238	−0.0210
3(C)	0.0288	0.0091	0.0189	−0.0197	3(C)	0.0165	0.0091	0.0128	−0.0074
4(C)	0.0211	0.0058	0.0135	−0.0153	4(C)	0.0324	0.0117	0.0220	−0.0207
5(C)	0.0184	0.0245	0.0215	0.0061	5(C)	0.0160	0.0083	0.0121	−0.0076
6(C)	0.0172	0.0253	0.0213	0.0081	6(C)	0.0319	0.0176	0.0247	−0.0143
7(C)	0.0331	0.0275	0.0303	−0.0056	7(C)	0.0970	0.1397	0.1183	0.0428
8(C)	0.0321	0.0285	0.0303	−0.0036	8(C)	0.0173	0.0143	0.0158	−0.0030
9(C)	0.0127	0.0261	0.0194	0.0134	9(C)	0.1898	0.1739	0.1819	−0.0159
10(C)	0.0356	0.0249	0.0302	−0.0106	10(C)	0.0231	0.0295	0.0263	0.0064
11(C)	0.0356	0.0272	0.0314	−0.0085	11(H)	0.0328	0.0242	0.0285	−0.0086
12(H)	0.0275	0.0517	0.0396	0.0242	12(H)	0.0174	0.0132	0.0153	−0.0041
13(H)	0.0268	0.0418	0.0343	0.0151	13(H)	0.0190	0.0153	0.0172	−0.0037
14(H)	0.0241	0.0325	0.0283	0.0084	14(H)	0.0179	0.0148	0.0163	−0.0031
15(H)	0.0266	0.0432	0.0349	0.0166	15(H)	0.0058	0.0020	0.0039	−0.0038
16(H)	0.0236	0.0334	0.0285	0.0098	16(H)	0.0314	0.0257	0.0286	−0.0058
17(H)	0.0258	0.0345	0.0302	0.0087	17(H)	0.0296	0.0218	0.0257	−0.0078
18(H)	0.0384	0.0533	0.0459	0.0149	18(H)	0.0313	0.0254	0.0284	−0.0059
19(H)	0.0261	0.0368	0.0314	0.0107	19(H)	0.0317	0.0296	0.0306	−0.0021
20(H)	0.0373	0.0543	0.0458	0.0170	20(H)	0.0228	0.0101	0.0165	−0.0127
21(H)	0.0192	0.0252	0.0222	0.0060	21(H)	0.0259	0.0300	0.0280	0.0040
22(H)	0.0190	0.0240	0.0215	0.0050	22(H)	0.0251	0.0200	0.0225	−0.0051
23(H)	0.0266	0.0407	0.0336	0.0142	23(H)	0.0574	0.0671	0.0623	0.0098
24(H)	0.0204	0.0207	0.0206	0.0003	24(H)	0.0590	0.0662	0.0626	0.0072
25(H)	0.0387	0.0400	0.0394	0.0014	25(H)	0.0297	0.0346	0.0322	0.0049
26(H)	0.0219	0.0261	0.0240	0.0043	26(H)	0.0375	0.0525	0.0450	0.0151
27(H)	0.0195	0.0232	0.0214	0.0038	27(O)	0.0291	0.0430	0.0360	0.0139
28(H)	0.0372	0.0428	0.0400	0.0056	28(H)	0.0020	0.0246	0.0133	0.0226
29(H)	0.0218	0.0283	0.0250	0.0065	29(H)	0.0174	0.0469	0.0322	0.0295
30(O)	0.0621	0.0425	0.0523	−0.0196					
31(H)	0.0241	0.0587	0.0414	0.0346					
32(H)	0.0057	0.0196	0.0127	0.0138					

**Table 5:** Chemical composition of essential oil of *P. bruguieri* plants (sample collection described in Section 2.1).

	Name	RI	RI Lit	RT	Area	Identification Method	Area%
1.	α-Pinene	936	939 [17]	5.141	240,990	RI, MS	2.76
2.	Camphene	952	952 [58]	5.330	19,190	RI, MS	0.22
3.	β-Pinene	980	981 [17]	5.684	22,704	RI, MS	9.63
4.	1-Octen-3-ol	987	-	5.813	56,885	RI, MS	0.65
5.	β-Myrcene	990	990 [59]	6.163	32,203	RI, MS	0.37
6.	α-Phellandrene	1004	1004 [59]	8.636	79,196	RI, MS	0.91
7.	Limonene	1029	1029 [59]	8.834	40,843	RI, MS	0.47
8.	1,8-Cineol	1095	1033 [59]	9.606	46,927	RI, MS	8.64
9.	γ-Terpinene	1060	1060 [59]	9.966	73,320	RI, MS	0.84
10.	Linalool	1145	1148 [60]	11.494	47,165	RI, MS	0.54
11.	Camphor	1185	1185 [60]	11.839	21,546	RI, MS	0.25
12.	Terpinen-4-ol	1205	1179 [59]	12.421	53,911	RI, MS	0.62
13.	Terpinolene	1210	1193 [59]	12.813	254,979	RI, MS	2.92
14.	Myrtenol	1216	1216 [60]	12.850	224,979	RI, MS	0.54
15.	Thymol	1297	1297 [61]	12.989	47,814	RI, MS	0.55
16.	Carvacrol	1300	1317 [61]	12.923	47,823	RI, MS	0.64
17.	α-Cubebene	1323	1337 [60]	13.140	47,270	RI, MS	8.89
18.	Eugenol	1345	1359 [59]	13.317	50,434	RI, MS	0.58
19.	α-Copaene	1352	1376 [61]	13.690	101,376	RI, MS	1.16
20.	δ-Cadinene	1358	1529 [59]	14.362	195,023	RI, MS	2.24
21.	β-Bourbonene	1388	1382 [62]	14.765	210,657	RI, MS	2.42
22.	Tiglate -3(Z)-hexenyl-	1390	1316 [63]	16.276	30,313	RI, MS	0.35
23.	β-Caryophyllene	1393	1392 [64,65]	33.265	111,541	RI, MS	8.28
24.	Capraldehyde	1400	1204 [66]	25.144	54,421	RI, MS	0.62
25.	Bicyclogermacrene	1443	1445 [60]	33.681	57,567	RI, MS	0.66
26.	α-Humulene	1418	1418 [60]	33.903	28,076	RI, MS	0.32
27.	Isobornil asetat	1467	-	34.636	33,822	RI, MS	0.39
28.	2-Methyl-4-pentenal	1466	-	40.941	46,066	RI, MS	3.08
29.	2-Hexen-1-ol	1470	1420 [67]	41.224	21,884	RI, MS	0.25
30.	Dodecanal	1477	1722 [68]	41.538	102,832	RI, MS	1.18
31.	Germacrene D	1490	1490 [60]	35.239	290,085	RI, MS	0.33
32.	Pentadecane	1502	1504 [69]	42.138	81,843	RI, MS	0.94
33.	Decanal	1504	1506 [68]	42.396	74,986	RI, MS	0.86
34.	Pentadecane	1510	1500 [69]	42.697	356,908	RI, MS	1.09
35.	γ-Cadinene	1514	1511 [61]	36.587	37,582	RI, MS	0.43
36.	Isolongifolene	1518	1517 [60]	36.775	84,774	RI, MS	0.97
37.	β-Selinene	1521	1441 [60]	36.986	81,178	RI, MS	0.93
38.	Lauric acid	1547	1547 [70]	43.814	23,501	RI, MS	0.27
39.	Germacrene B	1562	1524 [60]	37.247	50,873	RI, MS	0.58
40.	α-Curcumene	1569	1483 [61]	37.927	191,605	RI, MS	1.20
41.	Spathulenol	1572	1571 [61]	38.601	203,413	RI, MS	0.33
42.	Phthalate diethyl	1587	1587 [71]	52.791	101,397	RI, MS	1.16
43.	Caryophyllene oxide	1595	1578 [61]	39.221	93,680	RI, MS	10.56
44.	Undecane	1598	1100 [69]	42.214	720,462	RI, MS	1.91
45.	Nonadecane	1656	1900 [69]	53.812	84,321	RI, MS	0.97
46.	Phytone	1820	1827 [66]	51.922	308,615	RI, MS	3.54
47.	p-Cymen-8-ol	1850	1847 [66]	44.352	61,374	RI, MS	4.70
48.	Cetyl alcohol	1885	1882 [72]	52.480	56,068	RI, MS	0.64
49.	Heptadecyl alcohol	1980	1982 [73]	50.046	60,498	RI, MS	0.69
50.	2,2-Dimethyloctadecane	1910	1917 [74]	50.290	129,919	RI, MS	1.49
51.	Butylated hydroxytoluene	1920	1920 [69]	54.609	641,971	RI, MS	3.36
52.	Hexadecanoic acid, methyl ester	1930	1928 [75]	54.730	72,402	RI, MS	0.83
53.	Eicosane	2000	2000 [69]	57.202	66,550	RI, MS	0.76
54.	Heneicosane	2110	2100 [69]	48.955	56,028	RI, MS	0.49
Total							100.00

RI: retention index; RI Lit: retention index from the literature; RT: retention time. Identification methods: RI: based on retention index; MS: based on mass spectra matching.

## Data Availability

Not applicable.
